# Supplementary material for: Large three-dimensional photonic crystals based on monocrystalline liquid crystal blue phases
Source: Nat Commun. 2017 Sep 28;8:727. doi: 10.1038/s41467-017-00822-y (PMC5620071; doi:10.1038/s41467-017-00822-y)
Supplement: Supplementary file 1 — Supplementary Information [file 41467_2017_822_MOESM1_ESM.pdf]

Supplementary Note 1: Crosshatching and lattice orientation

Supplementary Figure 1a shows the crosshatching in a few ⟨011⟩-oriented BPI crystals of **M1**, in which the white solid and dashed lines describe, respectively, the striation directions and grain boundaries of crystals ① and ②. In each of the single crystals, the striations run in two directions, forming an obtuse angle. The bisector (dotted line) of the angle coincides with the ⟨200⟩ axis, confirmed by the Kössel diagram shown in Supplementary Fig. 1b. The lattice orientations are identified according to Supplementary Fig. 1c.[1] The free energies of such platelets are higher than their non-crosshatched counterparts because the crosshatching arose from the pseudomorphosis-induced tension. In this regard, the striations were found to be completely removed upon ~ 20 hours of relaxation.

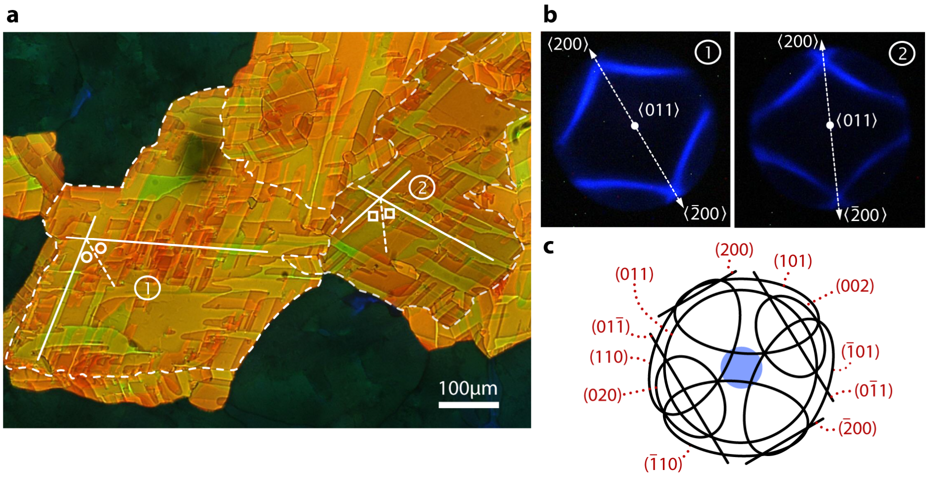

Supplementary Figure 1 | Correlation between crosshatching and lattice orientation. a, Microscope image of a few sub-mm-sized BPI crystals captured upon direct cooling from BPII. **b**, Observed Kössel diagrams for crystals ① and ②. **c**, Theoretical Kössel diagram for a BPI crystal viewed from ⟨011⟩. The blue region describes approximately the field of view of our experimental setup.

Supplementary Note 2: Estimation of BPI's reflection bandwidth

Yoshida *et al.*'s simulation results[2] suggest that the reflection bandwidth of a BPI crystal ($\Delta\lambda_{\mathrm{BPI}}$) is approximately one-fifth of that of a planar cholesteric liquid crystal ($\Delta\lambda_{\mathrm{N*}}$), on the assumption that the lattice spacings and material parameters are the same for both; more precisely, $\Delta\lambda_{\mathrm{BPI}} = 0.228 \times \Delta\lambda_{\mathrm{N*}}$. Therefore, we estimate the bandwidth of a single BPI crystal as follows: (i) Infiltrate the BPLC mixture into a surface-treated sandwich cell at a temperature above the clearing point, followed by cooling of the sample to the cholesteric phase. (ii) Store the sample at 25°C for almost a month to let the planar domains grow. (iii) Capture the transmission spectrum of a planar cholesteric domain (Supplementary Fig. 2). (iv) The bandwidth of a BPI crystal is estimated by substituting $\Delta\lambda_{\mathrm{N*}}$ with the measured bandwidth in:

$$\Delta\lambda_{\mathrm{BPI}} = 0.228 \times \Delta\lambda_{\mathrm{N*}} \frac{d_{hkl}}{p} = 0.228 \times \Delta\lambda_{\mathrm{N*}} \frac{\left(\lambda_{\mathrm{c}}/n_{\mathrm{avg}}\right)_{\mathrm{BPI}}}{\left(\lambda_{\mathrm{c}}/n_{\mathrm{avg}}\right)_{\mathrm{N*}}}, \tag{1}$$

where d_{hkl} is the lattice spacing along the [h k l] axis in BPI, p is the helical pitch in N*, λ_{c} is the center wavelength of the reflection band, and n_{avg} is the average refractive index along the propagation axis. For simplicity, by neglecting the difference between the average indices of BPI (square root of $(n_{\mathrm{e}}^2 + 2n_{\mathrm{o}}^2)/3$) and N* (square root of $(n_{\mathrm{e}}^2 + n_{\mathrm{o}}^2)/2$), Eq. (1) becomes:

$$\Delta\lambda_{\mathrm{BPI}} \cong 0.228 \times \Delta\lambda_{\mathrm{N^*}} \frac{\lambda_{\mathrm{c,BPI}}}{\lambda_{\mathrm{c,N^*}}}. \tag{2}$$

From the estimation, we obtain $\Delta\lambda_{\mathrm{BPI}} \approx 10.5$ nm for **M1** and $\Delta\lambda_{\mathrm{BPI}} \approx 13.8$ nm for **M2**, which are in good agreement with the experimental results. It is noteworthy that using the measured $\Delta\lambda_{\mathrm{N^*}}$ for such estimation may lead to some degree of inaccuracy in predicting $\Delta\lambda_{\mathrm{BPI}}$, owing to the bandgap broadening caused by imperfect planar alignment and/or inhomogeneous temperature distribution in the cholesteric.

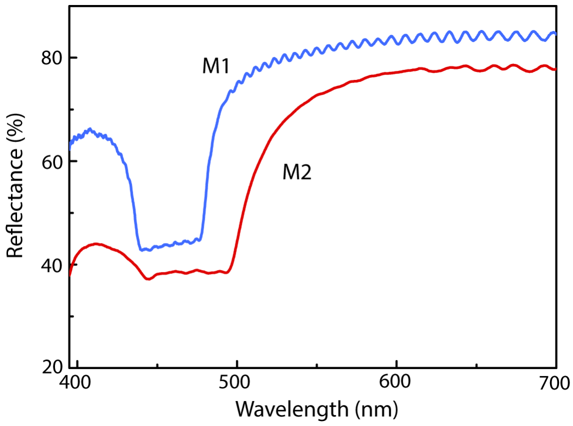

Supplementary Figure 2 | Estimation of BP's PBG from N*'s PBG. Transmission spectra of **M1** and **M2** in the (planar) cholesteric phase. Cell gaps $d \approx 20$ μm for the **M1** sample and $d \approx 100$ μm for the **M2** sample.

<u>Supplementary Note 3: Nearly mm^2-sized BP single crystal by self-reassembly</u>

Supplementary Figure 3 displays a nearly 1 mm^2-sized BPI monocrystal fabricated by maintaining a **M2** sample in the BPII phase for more than a week to let the polycrystal reassemble and then directly cooling down into the BPI phase. As observed under a polarizing optical microscope (Supplementary Fig. 3a), the terraces[3] become more closely spaced and the reflectivity drops near the convergent grain boundary (e.g. from ① to ② and from ① through ③ to ④). The corresponding Kössel diagrams in Supplementary Fig. 3b also imply the occurrence of macroscopic lattice deformation near the grain boundary; more precisely, a gradual inclination of the ⟨011⟩ axis towards the boundary. As for ⑤ and ⑥, the Kössel patterns indicate that they belong to an identical crystal with the orientation different from the neighboring (orange) ones.

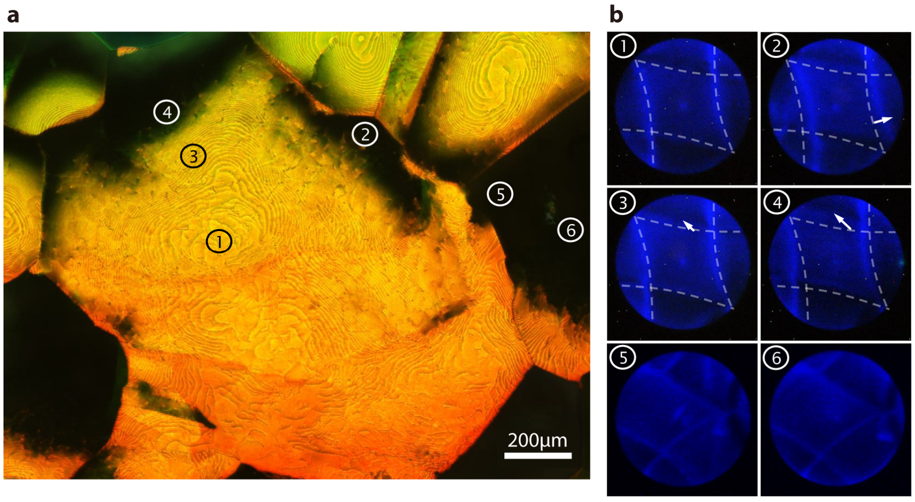

Supplementary Figure 3 | ~ 1 mm²-sized BPI monocrystal by self-reassembly in BPII & pseudomorphic transformation to BPI. a, Microscope image of the crystal. **b**, Kössel diagrams captured at labeled regions in **a**. The white dashed lines represent the Kössel diffraction pattern of ①.

Supplementary Note 4: Melt-and-regrow strategy

Supplementary Figure 4 demonstrates how the melt-and-regrow strategy can be adopted to extend the GTS crystal growth that has been terminated by unexpected fluctuations in temperature. By reversing the pulling direction, the grain boundaries of the BPI monocrystals can be erased, i.e. melted to BPII (Supplementary Fig. 4a,b). The growth process can subsequently be reactivated by reversing the pulling direction again (Supplementary Fig. 4c).

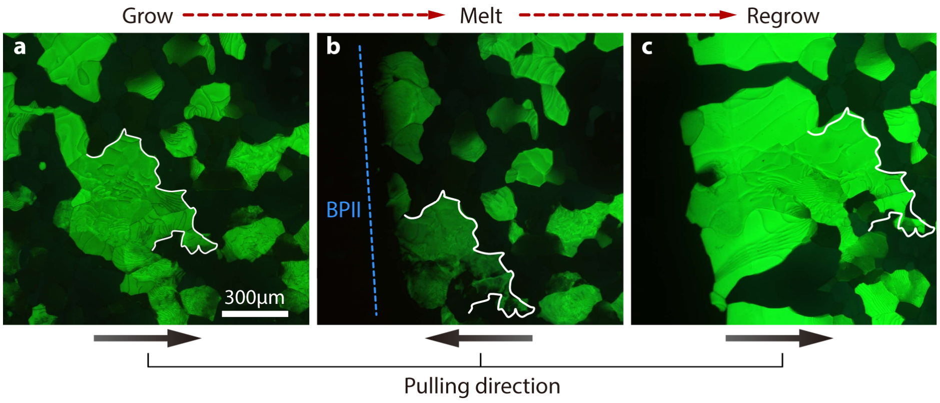

Supplementary Figure 4 | Melt and regrow. a, BPI monocrystals grown with a scan rate of 0.05 μm s^{-1}. The white solid line outlines part of a monocrystal for comparison with **b** & **c**. **b**, The grain boundaries of the BPI monocrystals are melt into BPII. The blue dotted line vaguely defines the interface of BPII and BPI. **c**, BPI monocrystals with extended length, regrown using a scan rate of 0.02 μm s^{-1}.

Supplementary Note 5: Electrical hysteresis and residual distortion of polymer-stabilized BP single crystal

Supplementary Figure 5 illustrates the electrical hysteresis loop of a polymer-stabilized BPI monocrystal by tracing the evolution of the PBG with increasing and decreasing direct-current field. The spectral positions of the PBG in the initial state and upon relaxation are both located at 559.9 nm, indicating that no residual distortion of the lattice is observed. The hysteresis, defined as the wavelength difference of the PBG at half tuning range between increasing and decreasing field, is ~ 2 nm. We believe that the hysteresis can be effectively eliminated by increasing the polymer content and optimize the photo-polymerization conditions to form a more robust polymer network.

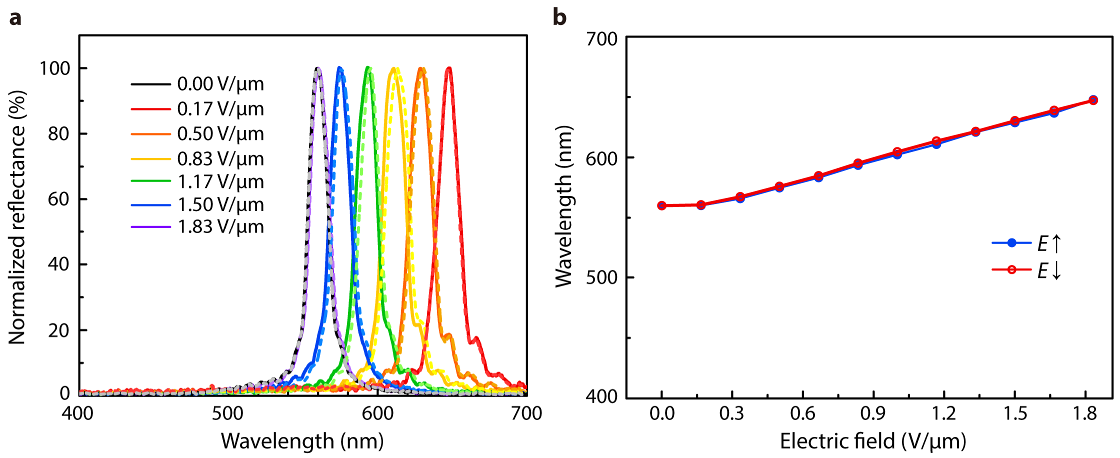

Supplementary Figure 5 | Electrical hysteresis and residual distortion. a, Reflection spectra at different direct-current field strengths. The solid lines and dashed lines correspond to the data collected with increasing and decreasing field, respectively. **b**, Electric field dependence of the peak reflection wavelength.

Supplementary References

1. Miller, R. J. & Gleeson, H. F. Lattice Parameter Measurements from the Kossel Diagrams of the Cubic Liquid Crystal Blue Phases. *J. Phys. II France* **6**, 909–922 (1996).
2. Yoshida, H. et al. Bragg reflection band width and optical rotatory dispersion of cubic blue-phase liquid crystals. *Phys. Rev. E* **94**, 042703 (2016).
3. Pierański, P., Barbet-Massin, R. & Cladis, P. E. Steps on surfaces of liquid-crystal blue phase I. *Phys. Rev. A* **31**, 3912-3923 (1985).
