# Supplementary material for: Large three-dimensional photonic crystals based on monocrystalline liquid crystal blue phases
Source: Nat Commun. 2017 Sep 28;8:727. doi: 10.1038/s41467-017-00822-y (PMC5620071; doi:10.1038/s41467-017-00822-y)
Supplement: Supplementary file 3 — Description of Additional Supplementary Files [file 41467_2017_822_MOESM3_ESM.pdf]

Description of Additional Supplementary Files

File Name: Supplementary Movie 1
Description: Self-reassembly dynamics of blue phase liquid crystals. Time-sequential microscope images comparing the platelet-merger rate in BPII with that in BPI.

File Name: Supplementary Movie 2
Description: Directional selective reflections from large blue-phase single crystals. Video of millimeter-sized BPI monocrystals illuminated from different angles. These crystals were grown by gradient-temperature scanning.

File Name: Supplementary Movie 3
Description: Growth dynamics of a centimeter-sized BPI single crystal during gradient-temperature scanning. Microscope video of a blue phase liquid crystal made in the process of surface-treatment assisted gradient-temperature scanning.
